# Exploring the association between pharmacy curricular tracks and students’ attitudes toward generic medicines: insights from a comparative study in Egypt

**DOI:** 10.3389/fphar.2026.1847196

**Published:** 2026-06-02

**Authors:** Eman. S. Sawan, Mahmoud Mostafa, Yara T. Sayed, Ammena Y. Binsaleh, Mahmoud A. Mohamed, Raghda R. S. Hussein

**Affiliations:** 1 Department of Clinical Pharmacy, Faculty of Pharmacy, Badr University in Cairo (BUC), Badr City, Egypt; 2 Faculty of Pharmacy, Badr University in Cairo (BUC), Badr City, Egypt; 3 Bioceutics Department, Faculty of Pharmacy, Tanta University, Tanta, Egypt; 4 Department of Pharmacy Practice, College of Pharmacy, Princess Nourah Bint Abdulrahman University, Riyadh, Saudi Arabia; 5 Hikma Pharmaceutical Company, Beni-Suef, Egypt; 6 Department of Clinical Pharmacy, Faculty of Pharmacy, Beni-Suef University, Beni-Suef, Egypt

**Keywords:** generic medicines, perceptions, Pharm D, Pharm D Clinical Pharmacy program, pharmacy educational tracks, pharmacy students

## Abstract

**Background:**

Use of generic medicines enhances drug affordability and accessibility. The aim if this study was to assess the association between pharmacy educational programs and final-year pharmacy students’ perceptions and confidence in generic medicines.

**Methodology:**

A cross-sectional survey was conducted in July 2025 among 248 fifth-year pharmacy students enrolled in two educational programs at Badr University in Cairo (BUC): the Pharm D Clinical Pharmacy program and the Pharm D program. The survey assessed knowledge of generic equivalency and beliefs regarding the quality, safety, and effectiveness of generic medicines compared to those of brand medications. Descriptive statistics, Chi-square test, and confidence intervals were used for data analysis, with a significance level set at p < 0.050.

**Results:**

Awareness of generic drug therapeutic equivalence was significantly higher in the Pharm D program than in the Pharm D clinical program (93.4% vs. 70.3%; p = 0.010; 95% CI: 0.02–0.10; Cramér’s V = 0.35). However, a notable proportion in both groups reported limited education regarding detailed bioequivalence issues. A substantial proportion of students in both programs reported the need for enhanced education about safety and efficacy of generics, especially in the clinical program (89.3% vs. 68.9%, p = 0.010; 95% CI: 0.02–0.10; Cramér’s V = 0.33).

**Conclusion:**

We identified significant differences in knowledge and attitudes regarding generic drugs between the two pharmacy programs. These differences are likely associated with variations in curriculum structure and content. Although general knowledge appears adequate, the Pharm D Clinical Pharmacy program may benefit from more targeted educational tracks about bioequivalence, quality assurance, and pharmacoeconomics that may better prepare future pharmacists to confidently recommend generic medications. Further multicenter studies are recommended to validate these findings.

## Highlights

- There were considerable differences between Pharm D and Pharm D Clinical Pharmacy students regarding perceptions of generics.
- Pharm D students were more aware of therapeutic equivalence than their counterparts.
- Many students reported insufficient education on bioequivalence concepts.
- There was a strong need for enhanced education about the safety and effectiveness of generics.
- A difference in curriculum might affect the ability of students to recommend generics.

## 1 Introduction

Global healthcare expenditures continue to escalate, with pharmaceutical costs representing 15%–30% of total healthcare budgets in most developing nations (Organization, 2024). Global pharmaceutical expenditures reached approximately 1.6 trillion USD in 2023. This reflects an increase of over $100 billion compared to 2022 (Frederick et al., 2025). Therefore, many nations, particularly developing countries, have adopted cost-control strategies to reduce their spending on pharmaceuticals (Leivaditis et al., 2025). Expanding the use of generic medications is one strategy for reducing costs without affecting the desired level of healthcare quality (Skarayadi et al., 2023). The effective application of such a strategy would lead to more competitive drug prices in the market and, ultimately, greater accessibility to affordable healthcare for a greater number of patients (Neumann et al., 2022).

Generic medications are produced by multiple manufacturers and marketed under the names of the active ingredients, while brand-name drugs are produced and supplied by a single company and marketed under a trade name (Aungaroon, 2023). The FDA necessitates a generic drug to have the same active ingredient, strength, dosage form, and route of administration as the brand drug. It must reveal bioequivalence by delivering the same amount of active ingredient into the bloodstream at the same rate and extent as the original brand drug. Additionally, generic drugs must show the same clinical effect and safety profile under labeled conditions to be considered therapeutically equivalent (Bell, 2022). Any variations must be restricted to the packaging and inert components such as flavoring, coloring, fillers, and stabilizing agents. Consequently, the FDA considers that approved generic and brand products can be substituted, with full expectation that the consumer will derive the same clinical benefit (Mukherjee, 2025). However, clinical practice recommended exercising caution and monitoring when prescribing narrow therapeutic index drugs, critical-dose medications, or highly variable drugs to ensure this clinical interchangeability (Gozzo et al., 2022; Luo et al., 2025). The use of FDA-approved generic drugs saved the American healthcare system $ 408 billion in 2022 (Tichy et al., 2022). Consequently, the FDA continued to rank generic pharmaceuticals as a top public health priority in 2022 (EDA, 2021). In Egypt, generic drugs are regulated by the Egyptian Drug Authority, which requires restricted quality and bioequivalence tests before generics enter the market, ensuring their quality, safety, and efficacy (EDA, 2021).

In fact, pharmacists play a crucial role in the healthcare system by providing patients and physicians with comprehensive drug information, managing drug interactions, in addition to counseling patients, recommending over-the-counter medications, and helping reduce prescription costs by suggesting generic substitutions (Kim et al., 2023).

Globally, many studies have documented misperceptions and doubts among pharmacy students, pharmacists, and healthcare providers regarding quality, safety, and efficacy of generic drugs, despite price discrepancies. In parallel, other studies indicated that it is often a challenging matter to change their perception and attitude toward generics (Al-Arifi, 2021; Wondrasek et al., 2024; Almas et al., 2025). However, increasing evidence suggests that improving healthcare providers’ awareness, through appropriate training and awareness programs, could boost their confidence and adherence to generic use (Desai et al., 2019). Consequently, studies that examine the pharmacy educational tracks and assess the viewpoints of current pharmacy students are recommended, especially in developing countries, to shape their knowledge and improve their perceptions toward generics (Al-Arifi, 2021). This approach could help detect various factors that influence professional behaviors and decision-making processes toward generic substitution, guiding the investigators to develop more tailored educational strategies to address the information gap about generic drugs (Qu et al., 2022; El-Jardali et al., 2017).

In Egypt, before 2019, certain pharmacy faculties provided the Pharm D degree as a postgraduate professional degree following completion of a five-year pharmacy bachelor’s degree (B Pharm). In 2019, the Egyptian Ministry of Higher Education (MoHE) and its pharmaceutical studies sector committee proposed replacing the current pharmacy bachelor’s degree (B Pharm) with a doctor of pharmacy degree (Pharm D–Drug Manufacture) or Pharm D Clinical Pharmacy. This modification was intended to ensure that the pharmacy education system is adequate for international standards. These programs typically consist of 5 years of academic education followed by 1 year of compulsory internship. This substantial reform was implemented countrywide across both public and private universities as it represents a primary qualification for pharmacy graduates in Egypt (Salem et al., 2022). Although the core structure of both curricula remains largely the same, differences in the depth of coverage and emphasis on specific topics may influence students’ knowledge, perceptions, and attitudes toward generic medications (Al-Saeed et al., 2026). The Pharm D program primarily covers essential pharmaceutical sciences, including pharmaceutics, pharmacology, medicinal chemistry, bioequivalence, pharmacoeconomics, and quality assurance, aimed at preparing students for a broad range of pharmacy roles, especially in pharmaceutical industries. On the contrary, the PharmD Clinical program offers deeper clinical experience through advanced therapeutics, patient-based care, and several clinical rotations in different healthcare settings (Salem et al., 2022).

Although former studies have investigated pharmacy students’ knowledge and attitudes toward generic medicines globally (Al-Arifi, 2021; Chaudhary et al., 2021), there remains a notable lack of research on pharmacy students across different educational programs in Egypt and the Middle East and North Africa (MENA) region. This gap limits the understanding of region-related factors that could influence students’ perceptions and future professional practices (Alqawasmeh et al., 2025). Some previous Egyptian studies highlighted persistent misconceptions regarding generic medicines, particularly in relation to their quality, safety, and therapeutic equivalence (ElHiny et al., 2021; Elmoneer, 2019). Additionally, recent research has underlined the need for continuous development of pharmacy education to better align with increasing healthcare demands (Al-Worafi and Al-Worafi, 2023).

Despite this existing evidence, comparative data evaluating the potential role of different pharmacy educational tracks in shaping students’ knowledge, perceptions, and attitudes toward generic medicines remain limited. Therefore, we aimed to bridge this identified gap through assessing knowledge, perceptions, and attitudes toward generic medications among final-year pharmacy students across two educational programs in Egypt.

## 2 Methods

### 2.1 The study sample

A survey-based descriptive cross-sectional comparative study was conducted among the fifth-year undergraduate pharmacy students in both programs, Pharm D Clinical Pharmacy and Pharm D, at BUC, enrolled in the 2024/2025 academic year. The survey was conducted in July 2025. Badr University in Cairo is a private university in Egypt, characterized by its innovation and academic excellence. According to the World’s Universities with Real Impact (WURI) 2025 rankings, it was the 33rd worldwide in innovation and is acknowledged in prominent international assessments such as the Shanghai and Times Higher Education rankings (WURI 2025). These achievements reflect the university’s commitment to quality education and scientific research (https://www.wuri.world).

Sample size calculation was performed using a Raosoft® calculator. The sample size was determined based on an estimated population of 169 students in the Pharm D clinical program and 98 students in the Pharm D program, with a predefined margin of error of 5%, a confidence level of 95%, and 50% response distribution, resulting in 118 and 79 individuals as the minimum recommended size for this survey for each group, respectively. Our sample sizes of 158 and 90 subjects, respectively, should, therefore, be sufficient to execute the survey. Raosoft Sample Size Calculator (2020) is available online at http://www.raosoft.com/samplesize.html.

### 2.2 Tools

To achieve the study objectives, an 18-item questionnaire was used to collect data from participants. These items had been validated in previous studies (Othman and Abdulghani, 2015; James et al., 2018) and were reviewed by two internal experts from the Clinical Pharmacy Department at BUC, namely, the first author and the department head, as well as researchers working with clinical pharmacy students in their final year, prior to submitting the protocol for ethical approval.

After participants were informed of the study’s purpose, written consent was requested. To guarantee a high response rate, consenting students in their classes were given questionnaires, which they were asked to return within 20 to 25 min. Participants were prohibited from using any kind of reference when responding to the questions. The questionnaire was administered using a paper-and-pen format. Every complete questionnaire was gathered and examined for accuracy.

The questionnaire was divided into four sections, which were distributed as follows: demographics (three items, age group, gender, and pharmacy program), awareness of generic equivalent drugs (four items), conceptions of students about quality, safety, and efficacy of generic medications versus brand name drug (eight items), and perceptions of generic medications in the current educational system (four items). Every question was scored using a 5-point Likert scale (Joshi et al., 2015), ranging from “strongly agree” to “strongly disagree,” except for the demographics question.

To validate the survey and to assess the reliability and clarity of the questionnaire, a pilot study involving 10 randomly selected final-year pharmacy students was carried out prior to the survey questionnaires being administered to the intended participants. Feedback from this pilot study helped the research team refine the questionnaire items to better align with the study goals. Results of this pilot study are an internal part of the current research and have not been separately published. Cronbach’s alpha, which was found to be 0.82, was used to determine reliability (Izah et al., 2023).

## 3 Statistical analysis

SPSS Version 26 for Windows (IBM, Armonk, New York, United States) was used to analyze the data. For each variable, descriptive statistics were computed, such as frequency distributions and percentages. The Chi-square test for categorical variables was used to evaluate the differences between the two groups (Pharm D Clinical and Pharm D). Confidence intervals (95% CI) were calculated for relevant comparisons to indicate the precision of the estimates. A 95% confidence interval (CI) represents a range of values within which the true value is likely to lie. In addition, Cramér’s V was calculated to assess the effect size of the observed associations (0.1 = small, 0.3 = moderate, and 0.5 = large).

## 4 Results

### 4.1 Demographic data

There were no statistically significant differences between Group 1 and Group 2 regarding age (p = 0.08; Cramér’s V = 0.09) or sex distribution (p = 0.07; Cramér’s V = 0.12). Demographic data of the study participants are shown in Table 1.

**TABLE 1:** Demographic comparison between pharm D clinical group (group 1) and pharm D group (group 2).

Characteristic	Group 1 (n = 158)	Group 2 (n = 90)	p-value	Cramér’s V
Age group	​	​	0.08	0.09
20–23 years	146 (92.4%)	78 (86.7%)	​	​
24–26 years	12 (7.6%)	12 (13.3%)	​	​
Sex	​	​	0.07	0.12
Male	45 (28.5%)	36 (40.0%)	​	​
Female	113 (71.5%)	54 (60.0%)	​	​
Participation %	​	​	0.40	​
​	93.5%	​	​	​
​	​	91.8%	​	​

Data are presented as a number (percentage). Group 1: Pharm D Clinical, Group 2: Pharm D. P-values were calculated using the Chi-square test to compare proportions between groups. A p-value < 0.05 was considered statistically significant. Cramér’s V was used to estimate effect size (0.1 = small, 0.3 = moderate, and 0.5 = large).

### 4.2 Awareness of generic equivalence compared to brand-name drugs

Both groups reported reasonable awareness of generic equivalence. Notably, 70.3% of students in Group 1 versus 93.4% in Group 2 agreed that generic medications are therapeutically equivalent to brand drugs (p = 0.01; 95% CI: 0.02–0.10; Cramér’s V = 0.35). However, 58.2% in Group 1 versus 33.3% in Group 2 (p = 0.04; Cramér’s V = 0.28) reported a misconception when they agreed that generic products are therapeutically equivalent to each other. Consistent with the recognized knowledge gaps, some students in both groups reported not being introduced to detailed bioequivalence concepts (32.9%) in Group 1 and (26.7%) in Group 2 (p = 0.04; 95% CI: 0.01–0.15; Cramér’s V = 0.26). Regarding the need for bioequivalence education, 62% of Group 1 reported the need for more structured knowledge, compared with 46.7% in Group 2. Although this difference did not reach statistical significance (p = 0.08; 95% CI: 0.03–0.14), the observed effect size suggests a small-to-moderate practical difference (Cramér’s V = 0.22). Students’ awareness of generic equivalence compared to brand-name drugs is summarized in Table 2.

**TABLE 2:** Awareness of generic equivalence compared to brand-name drugs.

Statement	Group	SA n (%)	A n (%)	N (%)	D n (%)	SD n (%)	p-value	95% CI	Cramér’s V
Generic products are therapeutically equivalent to brand-name products	Group 1 (n = 158)	39 (24.7%)	72 (45.6%)	20 (12.7%)	13 (8.2%)	14 (8.8%)	0.01	0.02–0.10	0.35
	Group 2 (n = 90)	60 (66.7%)	24 (26.7%)	2 (2.2%)	4 (4.4%)	0 (0.0%)	​	​	​
Generic products are therapeutically equivalent to each other	Group 1 (n = 158)	0 (0.0%)	92 (58.2%)	0 (0.0%)	66 (41.8%)	0 (0.0%)	0.04	0.01–0.12	0.28
	Group 2 (n = 90)	0 (0.0%)	30 (33.3%)	6 (6.7%)	54 (60.0%)	0 (0.0%)	​	​	​
Have not been introduced to detailed bioequivalence issues	Group 1 (n = 158)	0 (0.0%)	52 (32.9%)	26 (16.5%)	26 (16.5%)	54 (34.2%)	0.04	0.01–0.15	0.26
	Group 2 (n = 90)	24 (26.7%)	0 (0.0%)	12 (13.3%)	18 (20.0%)	36 (40.0%)	​	​	​
Need more information on bioequivalence tests	Group 1 (n = 158)	6 (3.8%)	92 (58.2%)	6 (3.8%)	39 (24.7%)	15 (9.5%)	0.08	0.03–0.14	0.22
	Group 2 (n = 90)	6 (6.7%)	36 (40.0%)	6 (6.7%)	0 (0.0%)	42 (46.6%)	​	​	​

Data are presented as a number (percentage). Group 1: Pharm D Clinical, Group 2: Pharm D. SA, strongly agree; A, agree; N, neutral; D, disagree; SD, strongly disagree. P-values were calculated using the Chi-square test to compare proportions between groups. A p-value < 0.05 was considered statistically significant. Cramér’s V was used to estimate effect size (0.1 = small, 0.3 = moderate, and 0.5 = large).

**FIGURE 1:**
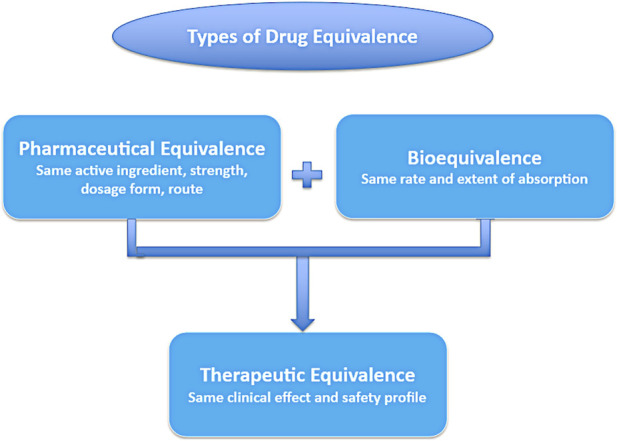
Conceptual relationship between types of drug equivalence. Pharmaceutical equivalence and bioequivalence together establish therapeutic equivalence, suggesting similar clinical efficacy and safety.

Actually, pharmaceutical equivalence means having the same active ingredient, strength, dosage form, and route. However, bioequivalence is to have the same rate and extent of absorption. Pharmaceutical equivalence and bioequivalence together establish therapeutic equivalence, suggesting similar clinical efficacy and safety. Different types of equivalence are illustrated in Figure 1.

### 4.3 Conceptions of students about quality, safety, and efficacy of generic medications versus brand-name drugs

Group 2 had a significantly higher belief that generics are bioequivalent (77.7%) than Group 1 (58.8%) (p = 0.01; 95% CI: 0.02–0.10; Cramér’s V = 0.41). Regarding the inferiority in quality, less effectiveness, and having more side effects of generic drugs compared to their brand-name counterparts, the majority of participants in both groups disagreed with these notions, with more prevalence in Group 2 than in Group 1 (80% vs. 74.7%, p = 0.03; 95% CI: 0.01–0.13; Cramér’s V = 0.25) concerning inferiority in quality, for example. Students’ conceptions about the quality, safety, and efficacy of generic medications compared to those of brand-name drugs are summarized in Table 3.

**TABLE 3:** Conceptions of students about quality, safety, and efficacy of generic medications versus brand-name drugs.

Statement	Group	SA n (%)	A n (%)	N (%)	D n (%)	SD n (%)	p-value	95% CI	Cramér’s V
A generic medicine is bioequivalent to a brand-name drug	Group 1 (n = 158)	19 (12.0%)	74 (46.8%)	47 (29.7%)	14 (8.9%)	4 (2.5%)	0.01	0.02–0.10	0.41
	Group 2 (n = 90)	66 (73.3%)	4 (4.4%)	18 (20.1%)	2 (2.2%)	0 (0.0%)	​	​	​
Generic medicine must be in the same dosage form	Group 1 (n = 158)	9 (5.7%)	74 (46.8%)	9 (5.7%)	57 (36.1%)	9 (5.7%)	0.02	0.01–0.11	0.38
	Group 2 (n = 90)	60 (66.7%)	2 (2.2%)	4 (4.4%)	24 (26.7%)	0 (0.0%)	​	​	​
Generic medicine must have the same dose as brand medicine	Group 1 (n = 158)	19 (12.0%)	66 (41.8%)	19 (12.0%)	47 (29.7%)	7 (4.5%)	0.03	0.02–0.12	0.36
	Group 2 (n = 90)	54 (60.0%)	6 (6.7%)	6 (6.7%)	24 (26.7%)	0 (0.0%)	​	​	​
Generic medicines are of inferior quality	Group 1 (n = 158)	6 (3.8%)	14 (8.9%)	20 (12.7%)	86 (54.4%)	32 (20.3%)	0.03	0.01–0.13	0.25
	Group 2 (n = 90)	6 (6.7%)	6 (6.7%)	6 (6.7%)	42 (46.7%)	30 (33.3%)	​	​	​
Generic medicines are less effective	Group 1 (n = 158)	17 (10.8%)	9 (5.7%)	28 (17.7%)	28 (17.7%)	76 (48.1%)	0.05	0.01–0.14	0.22
	Group 2 (n = 90)	6 (6.7%)	6 (6.7%)	6 (6.7%)	24 (26.7%)	48 (53.3%)	​	​	​
Generic medicines produce more side effects	Group 1 (n = 158)	17 (10.8%)	14 (8.9%)	28 (17.7%)	24 (15.2%)	75 (47.5%)	0.04	0.01–0.13	0.24
	Group 2 (n = 90)	6 (6.7%)	6 (6.7%)	6 (6.7%)	24 (26.7%)	48 (53.3%)	​	​	​
Generic medicines are less expensive	Group 1 (n = 158)	75 (47.5%)	57 (36.1%)	4 (2.5%)	7 (4.4%)	15 (9.5%)	0.01	0.02–0.10	0.34
	Group 2 (n = 90)	72 (80.0%)	2 (2.2%)	4 (4.4%)	12 (13.3%)	0 (0.0%)	​	​	​
Brand-name medicines meet higher safety standards	Group 1 (n = 158)	9 (5.7%)	74 (46.8%)	9 (5.7%)	9 (5.7%)	57 (36.1%)	0.02	0.01–0.12	0.27
	Group 2 (n = 90)	30 (33.4%)	24 (26.6%)	4 (4.4%)	2 (2.2%)	30 (33.4%)	​	​	​

Data are presented as a number (percentage). Group 1: Pharm D Clinical, Group 2: Pharm D. SA, strongly agree; A, agree; N, neutral; D, disagree; SD, strongly disagree. P-values were calculated using the Chi-square test to compare proportions between groups. A p-value < 0.05 was considered statistically significant. Cramér’s V was used to estimate effect size (0.1 = small, 0.3 = moderate, and 0.5 = large).

### 4.4 Conceptions of students about generic medications in the current educational system

When the final-year pharmacy students asked about their need for more information on safety and efficacy of generic medicines, 89.3% of Group 1 reported agreement compared to 68.9% in Group 2 (p = 0.01; 95% CI: 0.02, 0.10; Cramér’s V = 0.33). Additionally, the majority of participants in both groups reported their confidence in substituting brands, with higher confidence in Group 2 (p < 0.001; Cramér’s V = 0.37). Regarding the concept that it is easier to recall the generic-named drugs, moderate differences were also observed in perceptions (p = 0.02; Cramér’s V = 0.29). When asked about the coverage of cost-effective drug use in the pharmacy curriculum, 83.3% in Group 2 agreed compared to 76.5% in Group 1 (p = 0.03; 95% CI: 0.02–0.12, Cramér’s V = 0.31). Conceptions of participants about generic medications in the current educational system are displayed in Table 4.

**TABLE 4:** Conceptions of students about generic medications in the current educational system.

Statement	Group	SA n (%)	A n (%)	N (%)	D n (%)	SD n (%)	p-value	95% CI	Cramér’s V
Need more information on safety and efficacy of generics	Group 1 (n = 158)	66 (41.8%)	75 (47.5%)	9 (5.7%)	5 (3.2%)	3 (1.8%)	0.01	0.02–0.10	0.33
	Group 2 (n = 90)	60 (66.7%)	2 (2.2%)	0 (0.0%)	24 (26.7%)	4 (4.4%)	​	​	​
Confidence in substituting innovator brands	Group 1 (n = 158)	57 (36.1%)	80 (50.6%)	13 (8.2%)	5 (3.2%)	3 (1.8%)	0.00	0.02–0.09	0.37
	Group 2 (n = 90)	57 (63.3%)	18 (20.0%)	4 (4.4%)	11 (12.3%)	0 (0.0%)	​	​	​
Easier recall using generic names	Group 1 (n = 158)	61 (38.6%)	66 (41.8%)	3 (1.8%)	9 (5.7%)	19 (12.0%)	0.02	0.01–0.11	0.29
	Group 2 (n = 90)	60 (66.7%)	12 (13.4%)	4 (4.4%)	2 (2.1%)	12 (13.4%)	​	​	​
Pharmacy school covers the cost-effective use of generics	Group 1 (n = 158)	47 (29.7%)	74 (46.8%)	19 (12.0%)	14 (8.9%)	4 (2.5%)	0.03	0.02–0.12	0.31
	Group 2 (n = 90)	66 (73.3%)	9 (10.0%)	4 (4.4%)	2 (2.2%)	9 (10.0%)	​	​	​

Data are presented as a number (percentage). Group 1: Pharm D Clinical, Group 2: Pharm D. SA, strongly agree; A, agree; N, neutral; D, disagree; SD, strongly disagree. P-values were calculated using the Chi-square test to compare proportions between groups. A p-value < 0.05 was considered statistically significant. Cramér’s V was used to estimate effect size (0.1 = small, 0.3 = moderate, and 0.5 = large).

## 5 Discussion

Globally, generic medications have become a cornerstone of public health policy. The World Health Organization (2015) has called for increasing access to essential medicines and reducing healthcare expenses by increasingly making use of generic medications (Atif et al., 2019). Generic drugs can improve health conditions related to significant needs, particularly among people of low- and middle-income countries, due to a very high price barrier that has prevented them from accessing certain essential brand drugs (Yenet et al., 2023). However, misconceptions about generic drugs that are often propagated by healthcare professionals due to insufficient education could limit the effectiveness of national cost-containment policies. Consequently, strengthening pharmacy education could, therefore, support national policies promoting generic utilization and improve healthcare affordability and accessibility (Hatem et al., 2022; Al-Worafi, 2023).

In this study, we explore pharmacy education’s contribution to shaping the future pharmacists’ knowledge and attitude toward these drugs, a subject with limited research in Egypt. By comparing two pharmacy programs at Cairo’s Badr University, we identified substantial differences in attitudes and knowledge about generic drugs, emphasizing how curriculum design could be associated with differences in students’ self-confidence in prescribing cheaper alternatives to brand drugs.

Calamia and Abraham (2023) highlighted the continuing challenges that face generic promotion in many low-income countries. In these regions, persistent concerns regarding the quality and reliability of generic drugs can lead to a continued preference for brand drugs among healthcare professionals, including pharmacists, despite the lower cost of generics (Yenet et al., 2023; Calamia and Abraham, 2023). Therefore, it is essential to improve the conceptions of both the public and healthcare professionals about generics, safety, efficacy, and cost-effectiveness. Consequently, we aimed to explore the pharmacy students’ perceptions of generic medications and the existing knowledge gaps, highlighting the potential role of educational interventions in modulating such perceptions and gaps.

In the present study, there were no statistically significant differences in age or sex distribution between Group 1 and Group 2. However, the observed p-values were close to the significance threshold (age: p = 0.08; sex: p = 0.07), suggesting a potential minor imbalance. However, the effect sizes for these variables were small (Cramér’s V = 0.09 for age and 0.12 for sex), indicating that these differences are unlikely to have a meaningful impact on the study outcomes. The survey results showed significant differences with moderate-to-large effect sizes across several key variables, indicating meaningful differences between groups.

Both programs share a common basis of core pharmaceutical science courses, such as pharmacology, medicinal chemistry, pharmacognosy, microbiology, and pharmacokinetics. However, Group 2 showed better knowledge, perceptions, and attitudes toward generic medicines than Group 1. This may be related to the Pharm D curriculum structure, which mainly addresses the drug industry and pharmaceutical sciences. A clearer difference between the two programs is reflected in the distribution of drug-oriented courses and their corresponding credit hours (Cr H). Although both programs include core pharmaceutical subjects such as biopharmaceutics (3 Cr H in both programs) and marketing and pharmacoeconomics (2 Cr H in both programs), notable differences are observed in pharmaceutical and industrial courses. The Pharm D (Drug Manufacture) program allocates more credit hours to pharmaceutics (12 vs. 9 Cr H), industrial pharmacy (6 Cr H vs. 3 Cr H), quality control (3 Cr H vs. 2 Cr H), and quality assurance that is exclusively covered in the Pharm D program (2 Cr H vs. 0 Cr H) than in the Pharm D Clinical Pharmacy program.

However, the Pharm D Clinical Pharmacy program is more patient-oriented, incorporating multiple therapeutic modules, such as management of cardiovascular, gastrointestinal, dermatological, pediatric, neuropsychiatric, and oncological diseases, as well as endocrine and renal disorders, typically delivered as 3 Cr H each (with some modules, such as respiratory diseases and critical care, delivered as 2 Cr H). In contrast, the Pharm D (Drug Manufacture) program provides more limited clinical exposure, primarily through general courses such as clinical pharmacy I and II (3 Cr H each), hospital pharmacy (2 Cr H), and community pharmacy practice (3 Cr H), without equivalent disease-specific management courses. These differences may explain the relatively lower awareness of generic medicines in the Pharm D Clinical Pharmacy program, which includes several pharmacotherapy and disease management courses, limiting their exposure to bioequivalence and regulatory standards, while also promoting a more cautious, safety perspective that leads to a more critical evaluation of generic medicines.

Notably, 70.3% participants in Group 1 agreed that generic medicines are therapeutically equivalent to brand drugs compared to 93.4% in Group 2, which represents a strong understanding of the idea of therapeutic equivalence. This is in agreement with previous studies, which found that different healthcare programs influence awareness of generic medicines (Qu et al., 2022; El-Jardali et al., 2017).

The results of the present study revealed that many students in Group 1 and some in Group 2 (58.2% vs. 33.3%) agreed that generic products were therapeutically equivalent to each other. This further consolidates findings from Garcia et al. (2019) that a large number of students believe, incorrectly, that all products rated as “generic equivalents” are therapeutically equivalent to each other (Garcia et al., 2019). Two generic medicines may both be rated as equivalent to the brand-name drug, but they are not necessarily tested against each other. Therefore, they cannot be assumed to be therapeutically equivalent to each other without further studies. Manufacturing differences and different inactive ingredients in two different generic drugs can affect how the drug is absorbed or tolerated by the body. Additionally, the FDA allows generic drugs to have 80% to 125% of the bioavailability (absorption rate) compared to the brand-name drug. Therefore, one of these generic medicines might be at the low end (e.g., 80%) and the other at the high end (e.g., 125%), meaning that both can still be approved as a generic equivalent to the brand drug, although they may not be identical to each other (Bhushan and Martens, 2024; Noh et al., 2025).

The study participants reported some knowledge gaps regarding detailed training on bioequivalence issues during their studies in pharmacy, where 52 participants (32.9%) in Group 1 and 24 (26.7%) in Group 2 mentioned this claim. Such gaps could be addressed in the pharmacy curricula, especially in the clinical track, by incorporating specific bioequivalence, quality-assurance, and pharmacoeconomic considerations. Additionally, by using interactive lectures, online workshops, and training sessions with quality assurance experts that focus on regulatory limits and bioequivalence concepts, knowledge can be strengthened, confidence can be increased, and false beliefs among future healthcare providers can be corrected.

Evidence from a former study of Okoro et al. (2022) highlights a gap in the knowledge on bioequivalence among college students. Both groups had a high demand for information about bioequivalence tests, although Group 2 was more aligned with this statement than Group 1. The matter is associated with their expected future career as drug experts in the pharmaceutical industry. These students in the Pharm D program are expected to be involved in different aspects of drug production, formulation development, and quality control assessment compared to Pharm D Clinical students, aiming for clinical or patient-centered careers.

In parallel, a high number of students in Group 1 showed interest in acquiring information (62%), possibly because of less learning on this concept. Group 1, as future clinical pharmacists, also needs more knowledge regarding bioequivalence issues that are implemented in drug selection toward generics rather than brands.

Regarding the confidence toward the quality and efficacy conceptions, the present study reflected that Group 2 had a higher percentage of students than Group 1 (77.7% vs. 58.8%) who believed that a generic medicine is bioequivalent to a brand-name drug. This highly significant difference in response suggests that the Pharm D students are more exposed to pharmaceutical quality assurance, drug development processes, and policies. This background likely improves their confidence in generic medicines. However, clinical students may exhibit a more careful approach due to their training focus on patient safety. Previous studies have also depicted such beliefs related to bioequivalence and therapeutic equivalence (Calamia and Abraham, 2023; Bhushan and Martens, 2024; Noh et al., 2025).

Approximately one-half of both groups agreed that the generic medicine should be in the same dosage form and at the same dose as the brand-name medicine. It clearly reflects some misconceptions concerning specific criteria of generics manufacture, as found in studies by other research scholars, showing a lack of understanding in this area (Al-Mohamadi et al., 2018; Hassali et al., 2007). However, the majority of participants in both groups generally disagreed with the claim that generic medicines are of inferior quality, less effective, and have more side effects than brand medicines, indicating their support for generic replacement and their confidence in generic drugs as reliable treatment substitutes. This positive attitude reflects how their pharmacy curricula may emphasize the rigorous testing standards and regulatory requirements for generics to ensure their safety and efficacy compared to brands. By steadily integrating quality control topics in the coursework of pharmaceutics, pharmacology, and clinical pharmacy practice, the curricula may play a critical role in shaping the students’ perceptions and increasing the trust in generics among future pharmacists.

Concerning the generic medication conceptions in the present educational system, we demonstrated that a notably high proportion of students in both groups required additional information on safety and efficacy for generics, with a significantly higher percentage observed in Group 1 than in Group 2 (89.3% vs. 68.9%, p = 0.01). This may indicate a strong interest in acquiring more knowledge about this important concept. In addition, this may reflect some curricular gaps, which are parallel to the previous literature that identified such curricular gaps regarding bioequivalence and regulatory standards governing generic medicines within healthcare curricula (Tairabune et al., 2024). In addition, the majority of students in both groups felt confident about substituting brand-name drugs with generics. This finding is consistent with the study by Qu et al. (2022), which also highlighted that pharmacy students were generally confident in making generic substitutions (Qu et al., 2022). This confidence is crucial as it may refer to the students’ readiness to engage in cost-effective prescribing practices, which can significantly contribute to reducing healthcare costs and improving access to medications (Almangour et al., 2021). However, this high confidence in parallel with the noticed knowledge gaps suggests a potential disconnection between apparent competence and actual understanding, which was highlighted in earlier studies (Othman and Abdulghani, 2015; Al-Mohamadi et al., 2018).

Moreover, a high percentage of participants in Groups 1 and 2 (80.4% vs. 80.1%, respectively) reported that it was easier for them to remember the generic names. This could be related to the consistent use of generic nomenclature in pharmacy education, as supported by a recent study highlighting the role of academic exposure in shaping drug-related cognition (Slezáková et al., 2026).

In the same line, the majority of the participants in Groups 1 and 2 (76.5% vs. 83.3%, respectively) agreed that their pharmacy program has adequately covered the cost-effective use of generic medicines. This finding proposes that the Pharm D curriculum may place greater emphasis on pharmacoeconomics and cost–benefit relationships. This was an idea reported in a previous study by Tairabune et al. (2024), elucidating that the educational background influenced perceptions of generic medications (Tairabune et al., 2024). Additionally, these findings are in agreement with those of a former study that underscores the critical need for enhanced bioequivalence education in pharmacy curricula, particularly regarding quality control and regulatory standards (Almangour et al., 2021). This, therefore, means that there is considerable evidence from the previous literature supporting the current study regarding the presence of persistent knowledge gaps and negative perceptions about generic medicines. It further reiterates the need for improved educational strategies in healthcare programs to enhance use and acceptance of generic drugs, particularly with respect to bioequivalence and quality concerns.

Pharmacy education may play an important role in shaping the perceptions of future pharmacists on generic medicines. We elucidated that the limited educational exposure to bioequivalence concepts in the Pharm D clinical track seems to be associated with increased uncertainty and misconceptions regarding interchangeability and generic quality. These findings suggest that although clinical training is important, enhancing the educational content on specific bioequivalence, pharmacoeconomics, and quality assurance topics in the Pharm D clinical track may improve students’ overall understanding of generics. Although causality cannot be established here, the observed association suggests that limited bioequivalence education could be correlated with perceived quality gaps. This approach is in agreement with that by Kumari et al. (2023), who indicated that attitudes toward generics among pharmacy students were very much influenced by the curriculum they followed and the teaching methodology adopted (Kumari et al., 2023). Therefore, educational strategies should be updated to highlight generic characteristics such as bioequivalence testing, cost-effectiveness, and regulatory approval processes to ensure positive conceptions among pharmacy students (Neumann et al., 2022).

Finally, by incorporating more applied learning and clinical examples of effective generic drugs, pharmacy schools can increase confidence in using generics in practice (Othman and Abdulghani, 2015). Additionally, integrating manufacturing regulatory science and pharmacoeconomic considerations into clinical programs may improve the students’ understanding of bioequivalence and increase their confidence in generic substitution, thereby enhancing their future prescribing and dispensing practices (Al-Mohamadi et al., 2018).

## 6 Strengths

To the best of our knowledge, this is the first study in Egypt that addresses the potential association between different pharmacy programs and the final-year pharmacy students’ perceptions regarding generic medications. The well-structured comparative cross-sectional design allowed for a direct evaluation of differences between these educational tracks (Pharm D Clinical vs. Pharm D) within the same academic institution. The large sample size, high participation rate, and rigorous statistical analysis increased the validity of the observed data. Using a previously validated questionnaire, an initial pilot study for reliability (Cronbach’s alpha = 0.82) added more reliability to the findings. The consistency of moderate-to-large effect sizes across multiple variables strengthens the robustness of the observed differences and reduces the likelihood that these findings are due to random variation.

## 7 Limitations

However, this study has some limitations. The single institution setting may limit the generalizability to other educational institutions or countries with different curricular structures. Additionally, the cross-sectional nature of the study, lack of multivariate analysis, and deficient adjustment for confounders such as prior academic performance or work experience prohibit the interpretation that curriculum exposure alone is responsible for these results without longitudinal follow-up. Additional limitations include the reliance on self-reported data, which may introduce response or social desirability bias, as well as the reliance solely on quantitative data, rather than qualitative methods, such as open-ended questions or interviews, which could provide deeper insights into the underlying reasons behind their responses. Finally, the newly designed Egyptian pharmacy education system may be unfamiliar to some international readers who have a separate post-graduate Pharm D program.

## 8 Conclusion

Although most students seemed confident about the overall concepts of generics, misconceptions regarding their quality and bioequivalence still exist, underscoring the need for a more integrated educational approach that combines clinical training with comprehensive instruction in bioequivalence, regulatory standards, and pharmacoeconomics, especially in the Pharm D clinical program. This study provides a regional aspect addressing a global issue, assuming that an improved educational curriculum could be associated with greater acceptance of generic medications. Future multicenter studies with longitudinal follow-up, qualitative designs, and multivariate analyses are warranted to validate these findings across different Egyptian and MENA institutions and to elucidate more factors that could shape pharmacists’ confidence in generic medicines.

## Data Availability

The original contributions presented in the study are included in the article/supplementary material; further inquiries can be directed to the corresponding author.
